# Functional dissection of breast cancer risk-associated *TERT* promoter variants

**DOI:** 10.18632/oncotarget.18226

**Published:** 2017-05-26

**Authors:** Sonja Helbig, Leesa Wockner, Annick Bouendeu, Ursula Hille-Betz, Karen McCue, Juliet D French, Stacey L Edwards, Hilda A Pickett, Roger R Reddel, Georgia Chenevix-Trench, Thilo Dörk, Jonathan Beesley

**Affiliations:** ^1^ Gynaecology Research Unit, Hannover Medical School, Hannover, Germany; ^2^ Department of Genetics and Computational Biology, QIMR Berghofer Medical Research Institute, Brisbane, Australia; ^3^ Statistics Unit, QIMR Berghofer Medical Research Institute, Brisbane, Australia; ^4^ Telomere Length Regulation Unit, Children's Medical Research Institute, University of Sydney, Westmead, Australia; ^5^ Cancer Research Unit, Children's Medical Research Institute, University of Sydney, Westmead, Australia

**Keywords:** telomerase, SNP, breast carcinoma, GABPA, GWAS

## Abstract

The multi-cancer susceptibility locus at 5p15.33 includes *TERT*, encoding the telomerase catalytic subunit. Genome-wide association studies (GWAS) have identified six single nucleotide polymorphisms (SNPs) in the *TERT* promoter associated with decreased breast cancer risk, although the precise causal variants and their mechanisms of action have remained elusive. Luciferase reporter assays indicated that the protective haplotype reduced *TERT* promoter activity in human mammary epithelial and cancer cells in an estrogen-independent manner. Using single variant constructs, we identified rs3215401 and rs2853669 as likely functional variants. Silencing of MYC decreased *TERT* promoter activity but neither MYC nor ETS2 silencing conferred allele-specificity. In chromatin immunoprecipitation experiments, the ETS protein GABPA, but not ETS2 or ELF1, bound rs2853669 in an allele-specific manner in mammary epithelial cells. Investigation of open chromatin in mammoplasty samples suggested involvement of three additional variants, though not rs3215401 or rs2853669. Chromosome conformation capture revealed no interaction of the *TERT* promoter with regulatory elements in the locus, indicating limited local impact of candidate variants on the *TERT* promoter. Collectively, our functional studies of the *TERT*-*CLPTM1L* breast cancer susceptibility locus describe rs2853669 as a functional variant of this association signal among three other potentially causal variants and demonstrate the versatile mechanisms by which *TERT* promoter variants may affect breast cancer risk.

## INTRODUCTION

Telomeres consist of DNA tandem repeats and telomere binding proteins, which together form secondary nucleoprotein structures at the end of chromosomes [1, 2]. In humans, telomeres are 10-15 kilobase pairs long with a double stranded sequence repetition of TTAGGG followed by a G-rich, single stranded 50-300 nucleotide extension named G-strand overhang [3, 4]. Although telomeres are highly conserved, variation is intrinsic to the repeated sequence, the hetero- or homogeneity of repeats and their length [1, 3, 5].

A major function of telomeres is to prevent the ends of chromosomes from being recognized as DNA strand breaks and therefore to protect against genomic instability. Also, due to the “end-replication problem”, the ends of linear DNA molecules are not fully replicated, and this limits the number of divisions a cell can undergo before entering the state of permanent cell cycle arrest referred to as senescence [6–10]. Therefore, another function of telomeres is to act as a buffer against the loss of coding or regulatory DNA during cell proliferation. When cells bypass senescence and continue to proliferate, telomeres become shortened to a critical length leading to a state of crisis with chromosomal end-to-end-fusion events and genomic instability [11, 12].

Some cell types such as stem and germline cells maintain a constant telomere length [12–15]. In these cell types telomerase elongates telomeres and thereby increases the proliferative capacity [16, 17]. In most normal tissues telomerase is absent or present at low levels, allowing natural cellular aging. In contrast, cancer cells can avoid cellular senescence by spontaneously activating a telomere lengthening mechanism, such as telomerase [18, 19]. In 90% of cancers, telomerase activity is significantly increased but the molecular mechanisms behind its activation have not been fully resolved [20, 21].

Telomerase contains a protein catalytic subunit with reverse transcriptase activity, TERT, and an RNA subunit, TERC [16, 22, 23]. TERT is a limiting factor of telomerase activity and its regulation mainly occurs at the transcriptional level [24, 25]. The *TERT* upstream region includes many transcription factor consensus sequences and a CpG island. Presumably, these characteristics contribute towards its tightly regulated transcriptional activity [26]. Many regulators of *TERT* promoter activity have been reported, such as transcriptional activators (MYC, ETS2, SP1, HIF1A and AP2) and repressors (AP1, EGR1, MEN1 and WT1), and sex hormones (estrogen and androgen) [27–40].

GWAS have identified the *TERT-CLPTM1L* region on chromosome 5p15.33 as a breast cancer susceptibility locus with three independent genetic signals, which comprise variants in strong linkage disequilibrium [41]. The strongest signal, signal 1, covers the *TERT* promoter and harbors six closely correlated candidate variants: rs2736107, rs2736108, rs2736109, rs145544133 (synonym: rs10548207), rs3215401 and rs2853669, which for simplicity will be designated variants 1-6 in the figures and Table 1. The variants rs145544133 and rs3215401 are insertion/deletion polymorphisms (indels). The risk-associated alleles of the six candidate variants are associated with a decreased risk of overall breast cancer, estrogen receptor (ER) positive breast cancer and breast cancer in *BRCA1* mutation carriers but most strongly with ER-negative breast cancer [41]. Previous studies of these six variants in breast cancer cell lines indicated that the risk-associated alleles of variants rs2736107, rs2736108 and rs2736109 decrease *TERT* promoter activity *in vitro*, whereas rs2853669 had no effect [41, 42].

**Table 1:** *TERT* promoter variants with predicted change of regulatory motifs

SNP	rs ID	hg19 position	Major/risk-associated allele	MAF	Regulatory motifs
SNP1	rs2736107	1,297,854	G/A	0.27	ESR1, GATA, RXRA
SNP2	rs2736108	1,297,488	G/A	0.28	HENMT1, ZBTB14
SNP3	rs2736109	1,296,759	G/A	0.34	GATA, NR3C1, MAF, SIX5
SNP4	rs145544133 (rs10548207)	1,297,078	CC/-	0.28	BDP1, CACYBP, CCNT2, EGR1, TRIM63, KLF4, KLF7, PATZ1, MYC, PPARA, PAX4, POU2F2, RREB1, SP1, ZMIZ, UF1H3BETA, ZNF219, ZNF281, ZNF740,
SNP5	rs3215401	1,296,255	-/C	0.30	SP2, ZBTB7A
SNP6	rs2853669	1,295,349	T/C	0.30	ETS, MYC, MIXL1, RBPJ, SIN3A, ZNF143, EP300

*TERT* promoter polymorphisms were designated SNPs 1-6 as indicated. Their location on chromosome 5, their major /risk-associated alleles and their major allele frequency (MAF) were drawn from the 1000 Genome browser based on Ensembl v80 GRCh37. Data on regulatory motifs affected by the respective variants were acquired through HaploReg v3.

Due to their location in the promoter, the risk variants are hypothesized to regulate *TERT* transcription, and are predicted to alter numerous transcription factor (TF) binding motifs (Table 1). Variant rs2736107 resides in an estrogen responsive element (ERE) [38], while rs2853669 is located in an ETS consensus sequence adjacent to an E-box motif specific for MYC binding [26]. A previous report indicated combined activation of *TERT* expression by MYC and ETS2 at this location in breast cancer cells [28]. In addition, recurrent somatic mutations have been reported, which create novel ETS binding sites in the *TERT* promoter that are associated with an increased *TERT* expression in different cancers [43–45]. These novel ETS motifs act like a regulatory switch [46], which open the chromatin and allow the transcription factor GABP to activate *TERT* transcription [46]. Somatic mutations in ETS binding sites may interact with rs2853669 to influence mortality from some cancers [47–51]. In addition to TFs, long-range chromatin interactions between distant regulatory elements and promoters facilitate regulation of gene expression by chromatin looping, and some breast cancer risk variants at other loci have been shown to influence this process [51, 52].

The aim of the present study was to define the effect of the risk-associated haplotype in the *TERT* promoter and to identify the causal variant(s) underlying its association with breast cancer risk.

## RESULTS

### The risk-associated haplotype of rs3215401 and rs2853669 reduced TERT promoter activity

**Figure 1:**
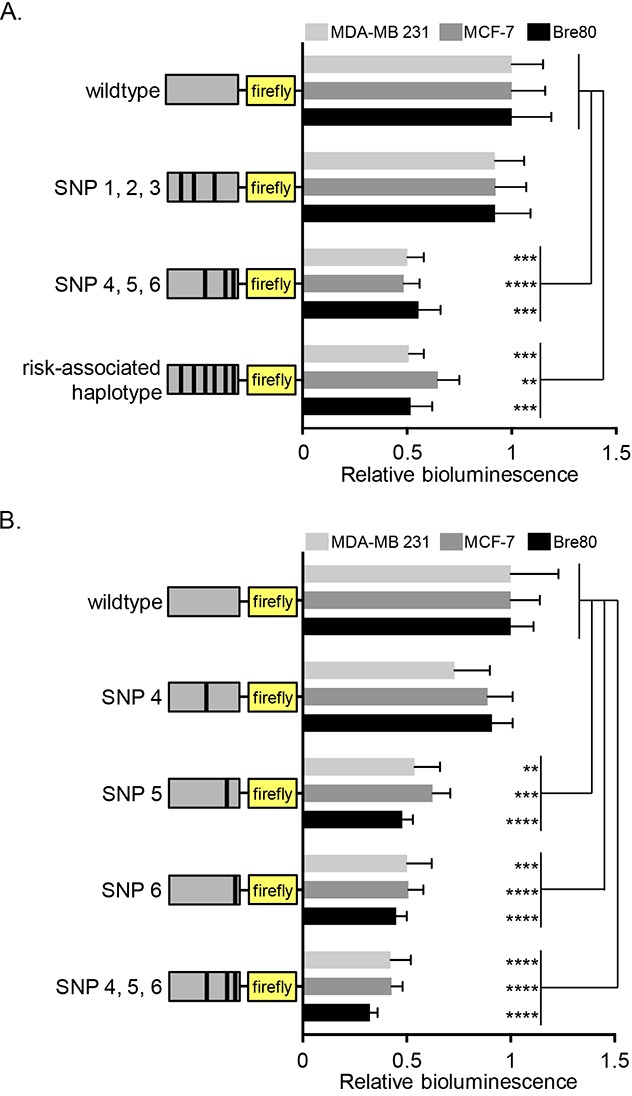
Effect of protective variants on *TERT* promoter activity in mammary epithelial and breast cancer cell lines Cell lines were transfected with *TERT* promoter reporter constructs carrying risk alleles of the six candidate variants as indicated in the graph. **(A)** Comparison of wildtype and risk-associated haplotypes as well as the partial haplotypes carrying risk-associated alleles of SNPs 1-3 (rs2736107, rs2736108, rs2736109) or SNPs 4-6 (rs145544133, rs3215401, rs2853669), respectively. **(B)** Comparison of wildtype with single variant haplotypes for SNP4 (rs145544133), SNP5 (rs3215401), or SNP6 (rs3215401), and the partial SNP 4-6 (rs145544133, rs3215401, rs2853669) haplotype. Values were normalized to wildtype. The figures represent the estimated marginal effect (with 95% confidence interval) of data from at least 3 experiments performed on separate days. To account for the average difference between separate days a two-way ANOVA was performed with experiment day as a block variable. P-values from post-hoc comparisons between WT and the other groups were adjusted using Dunnett's correction (* P<0.05, ** P<0.01, ***P<0.001 ****P<0.0001). All statistical analyses were performed in log-scale, values were back-transformed for plots presented. An empty pGL3Basic vector was transfected into cells as negative control showing that *TERT* promoter activity is above background (Supplementary Figure 2).

The risk-associated alleles of rs2736107, rs2736108 and rs2736109 have previously been reported to decrease *TERT* promoter activity [41, 42], although the underlying mechanism has remained elusive. We generated luciferase reporter constructs containing 3.9 kb of the *TERT* promoter with either all the protective alleles (wildtype), or all risk-associated alleles of the six candidate variants (risk-associated haplotype). Luciferase assays showed that the risk-associated haplotype reduced *TERT* promoter activity significantly in Bre80 normal breast cells, ER-positive MCF-7 and ER-negative MDA-MB-231 breast cancer cell lines (Figure 1A).

We further divided the constructs into haplotypes comprising either the risk-associated alleles of rs2736107, rs2736108, rs2736109 or of rs145544133, rs3215401, rs2853669. Luciferase assays showed that *TERT* promoter activity was significantly decreased with risk-associated alleles of the last three variants (rs145544133, rs3215401, rs2853669: labeled SNP4, 5, and 6, respectively, in the figure), though not with the first three (rs2736107, rs2736108, rs2736109, labeled SNP1, 2, and 3, respectively, in the figure). To clarify the roles of rs145544133, rs3215401 and rs2853669, we generated single variant constructs harboring individual risk-associated alleles. There was no detectable effect of rs145544133 but significant reduction of *TERT* promoter activity with either rs3215401 or rs2853669 (Figure 1B).

Since these data were inconsistent with earlier studies indicating rs2736107, rs2736108 and rs2736109, but not rs2853669, as variants that reduced *TERT* promoter activity in breast cancer cells [41, 42], we performed Sanger sequencing of the previously used constructs. This revealed 11 additional variants (including common polymorphisms) of unknown significance compared with the human reference genome sequence (Supplementary Table 2). When we performed comparative luciferase reporter assays using the constructs with and without these 11 variants in parallel, we were able to replicate both our present results, as well as the results previously published (Supplementary Figure 3) [41, 42]. This indicates that the additional variants present in the constructs used in previous studies [41, 42] might have influenced the outcomes of luciferase assays for rs2736107, rs2736108, rs2736109 and rs2853669. The variants rs145544133 and rs3215401 had not been assayed previously.

### Allele-specific effects on TERT promoter activity are independent of MYC, ETS2 and estrogen

**Figure 2:**
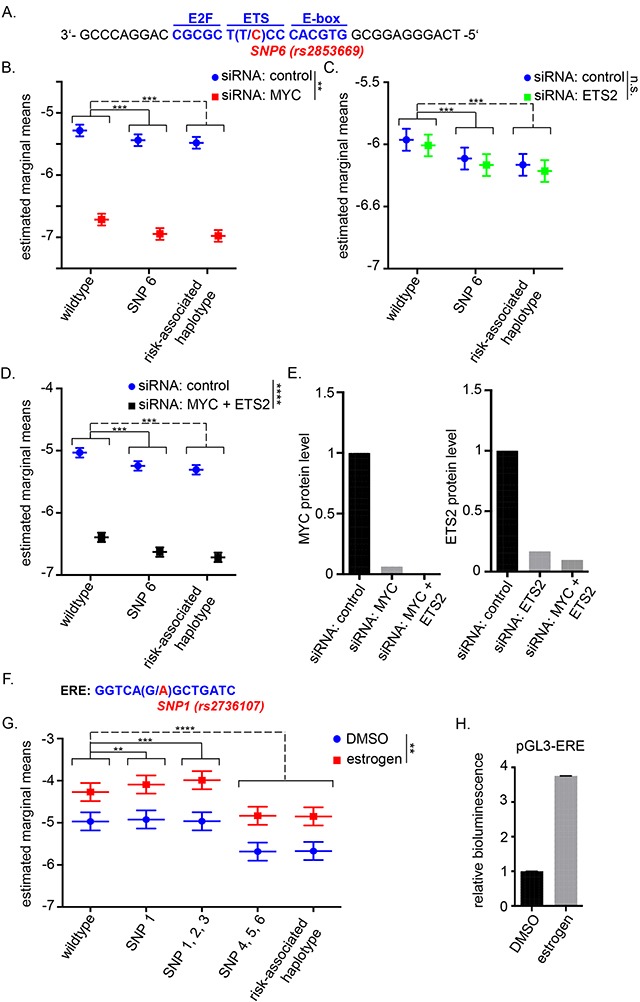
*TERT* promoter activity upon MYC and/or ETS2 silencing and estrogen induction **(A)** Schematic location of SNP6 (rs2853669, major allele T/risk-associated allele C) in the *TERT* promoter with 3 major transcription factor binding sites: E2F, ETS and E-box. **(B, C, D)** ER-negative (ER-) MDA-MB 231 cells were transfected with siRNA then 24h later transfected with *TERT* promoter constructs. The wildtype construct was compared to the construct carrying the risk-associated allele of SNP6 (rs2853669) and the risk-associated haplotype after treatment with control siRNA or MYC targeting siRNA **(B)**, ETS2 targeting siRNA **(C)** or MYC and ETS2 targeting siRNA **(D)**. Asterisks next to the legend illustrate the significance of the overall interaction between control siRNA and MYC and/or ETS2 targeting siRNA. Similar data were obtained for ER-positive (ER+) MCF-7 cells and Bre80 normal breast cells (Supplementary Figure 4A, 4B). **(E)** Knock-down efficiency was determined by western blot analysis and was quantified for one experiment by ImageJ (western blots are shown in Supplementary Figure 4C). **(F)** Position of SNP1 (rs2736107) in the estrogen responsive element of the *TERT* promoter. **(G)** ER+ MCF-7 cells were cultured in phenol-red free media and treated with 10nM fulvestrant for 48h. Afterwards, cells were transfected with *TERT* promoter reporter constructs carrying major (wildtype) alleles or risk-alleles as indicated. The medium contained 20nM estrogen or DMSO as a control during and after transfection. The wildtype construct was compared to that carrying the risk-associated allele of SNP1 (rs2736107) alone, the partial haplotypes carrying risk-associated alleles of SNPs 1-3 (rs2736107, rs2736108, rs2736109) or SNPs 4-6 (rs145544133, rs3215401, rs2853669), respectively, and the risk-associated haplotype carrying all six risk-associated alleles. **(H)** Estrogen induction was verified by pGL3 vector containing an estrogen responsible element. Data are shown for at least three experiments **(B, C, G)** or two experiments **(D)**. Three-way ANOVA was used to assess the effect of group, estrogen/siRNA and the interaction of group and estrogen/siRNA. Experiments were performed on separate days and results were combined by including day as a blocking factor into the ANOVA. The figures represent the estimated marginal effect (with 95% confidence interval) of each treatment combination after accounting for the average difference between separate days. Multiple comparisons between groups of interest were defined via contrasts. P-values were adjusted using Bonferroni's multiple hypotheses testing adjustment (** P<0.01, ***P<0.001 ****P<0.0001). All statistical analysis was performed in log-scale.

The candidate causal variant rs2853669 resides in one of two ETS binding sites in the *TERT* promoter and is adjacent to an E-box motif, the consensus sequence for MYC binding (Figure 2A). ETS2 reportedly activates *TERT* expression in cooperation with MYC at this particular site [28, 53]. We therefore investigated the effect of siRNA mediated knock-down of ETS2 and MYC on the *TERT* promoter activity of our haplotype constructs in Bre80, ER-negative MDA-MB-231, ER-positive MCF-7 cell lines. Regardless of the rs2853669 genotype or haplotype, silencing of MYC resulted in the expected down-regulation of *TERT* promoter activity (Figure 2B; Supplementary Figure 4). Unexpectedly, silencing of ETS2 showed no significant effect on *TERT* promoter activity (Figure 2C; Supplementary Figure 4). As ETS2 and MYC may cooperatively bind to their consensus sequences, we performed a double knock-down of MYC and ETS2. Luciferase assays after combined silencing gave results similar to the silencing of MYC alone (Figure 2D; Supplementary Figure 4). Successful silencing of MYC and ETS2 protein was confirmed by western blot (Figure 2E; Supplementary Figure 4). These results indicated that the effect of rs2853669, located in the ETS binding site, was not dependent on the levels of ETS2 under our experimental conditions.

Since telomerase activity and *TERT* expression are inducible upon estrogen administration and rs2736107 resides within a previously identified estrogen responsive element (ERE) (Figure 2F, [38]), we also tested the effect of estrogen treatment on the wildtype promoter and the *TERT* promoter harboring the risk-associated allele of rs2736107 by means of luciferase reporter assays in the ER-positive MCF-7 cells. Estrogen supplementation markedly induced promoter activity in this assay. The induction was slightly altered by rs2736107 and by the partial haplotype rs2736107, rs2736108, rs2736109 compared to wildtype but this differential effect was non-significant (Figure 2G, 2H).

### Allele-specific binding of GABPA and MYC to rs2853669

**Figure 3:**
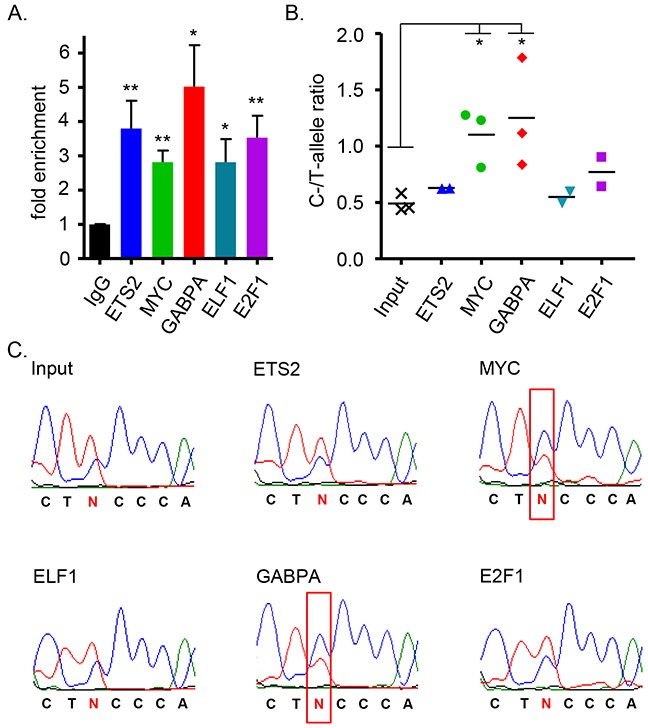
Allele-specificity of transcription factor binding at the site of rs2853669 (SNP6) **(A)** Chromatin immunoprecipitation (ChIP) enrichment was determined by qRT-PCR using ETS2 (n=2), E2F1 (n=2), ELF1 (n=2), MYC (n=3), GABPA (n=3) antibodies for pulldown of genomic region surrounding SNP6 (rs2853669) in the heterozygous normal mammary cell line Bre80. For comparison the unpaired, two-tailed t-test was used (*P<0.05, **P<0.01). **(B)** Quantitative data of C- to T-allele ratio after ChIP enrichment were generated using the mean sequencing peak heights. A Bre80 input sample was included as reference. For comparison the unpaired, two-tailed t-test was used (*P<0.05). **(C)** Representative sequencing data of one of three independent experiments are presented. Red box indicates switch of allele ratio.

The ETS family of transcription factors has 27 members, and apart from the reported role of ETS2 in *TERT* expression [28, 53], the transcription factors GABPA and ELF1 have also been implicated to bind at native and de novo ETS binding sites, possibly regulating *TERT* transcription [54, 55]. Neighboring the ETS binding site is the consensus sequence for E2F1 (Figure 2A), a transcription factor reported to bind exclusively the major allele of rs2853669 in a hepatic cancer cell line [56]. We therefore considered E2F1, ELF1 and GABPA in addition to ETS2 and MYC as candidate transcription factors acting through the rs2853669 site and performed chromatin immunoprecipitation (ChIP) in Bre80 cells (Figure 3A), which are considered “normal” to represent normal breast tissue and are heterozygous for the six risk-associated candidate variants. There was no discernable difference in the allelic ratios between the input control sample and the ChIP sample for ETS2, ELF1, or E2F1. In contrast, GABPA and MYC ChIP led to the preferential isolation of the risk-associated C allele of rs2853669 (Figure 3B, 3C), indicating GABPA as the only one of the three tested ETS proteins with allele-specific binding to the rs2853669 site in Bre80 cells.

### Human TERT promoter does not interact with other regions at the TERT-CLPTM1L locus but rs2736108 and rs2736109 are differentially associated with chromatin accessibility

Since long-range DNA-DNA interactions play an important role in the regulation of gene expression, we also asked whether the effect of the protective haplotype is restricted to local *TERT* promoter activity or whether this region may be involved in distal chromatin looping. Chromosome conformation capture (3C) assays were performed to detect interactions between the *TERT* promoter and NcoI fragments between hg19 co-ordinates chr5: 1,249,069-1,358,885 in Bre80 and MCF-7 cells.

Using the *TERT* promoter as bait, we found one interaction peak that suggested chromatin looping between the *TERT* promoter and the *CLPTM1L* promoter in both cell lines (Supplementary Figure 5A, 5C). However, this interaction was not allele-specific (Supplementary Figure 5E, 5F) and a reciprocal validation experiment using the *CLPTM1L* promoter as bait failed to validate chromatin looping (Supplementary Figure 5B, 5D). High interaction frequencies were only detected for fragments adjacent to baits reflecting the expected pattern due to fragment proximity.

To investigate the influence of *TERT* promoter variants on chromatin accessibility, we performed formaldehyde-assisted isolation of regulatory elements (FAIRE) on 22 breast tissue samples, followed by genotyping using allele-specific SNP-type assays. In the heterozygous samples (8/22), FAIRE-enriched samples of rs2736108 and rs2736109 displayed a shift to their major alleles, (G for both variants; Supplementary Figure 6A, 6B). However, rs3215401 and rs2853669 demonstrated little change in pattern after FAIRE-enrichment indicating lack of association with open chromatin (Supplementary Figure 6C, 6D). These results indicated inconsistent patterns of allele-specific chromatin accessibility across the *TERT* promoter in primary breast tissue.

## DISCUSSION

GWAS have been very successful in identifying cancer susceptibility loci, but to pin-pointing the causal variants, among many correlated candidate variants, usually requires subsequent *in silico* annotation and functional studies [57]. We applied several methods to investigate the breast cancer risk associated variants at the *TERT-CLPTM1L* region, a known multi-cancer risk locus [44, 45, 47, 49, 50, 58–60]. Risk-associated alleles in the *TERT* promoter are associated with decreased overall breast cancer risk and the association is most significant with ER-negative breast cancer [41]. As they are primarily located in the *TERT* promoter, we hypothesized that the variants influence *TERT* transcription. Our reporter assays showed that the risk-associated haplotype significantly reduced *TERT* promoter activity, and this effect could be attributed to rs3215401 and rs2853669. Although previous literature presented variants rs2736107, rs2736108 and rs2736109 but not rs2853669 as variants effective in reducing *TERT* promoter activity [41, 42], we now show that this result was probably influenced by non-breast cancer risk associated variants present in the constructs used in prior studies. (Supplementary Figure 3).

**Figure 4:**
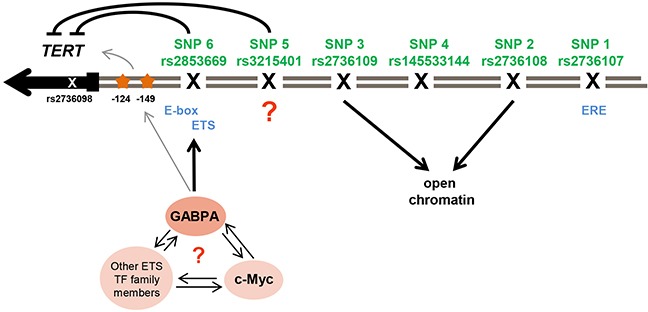
Illustration of potential regulation of *TERT* transcription and chromatin configuration by its promoter SNPs The six promoter SNPs that have been analyzed (in green with rs-number) and the SNP rs2736098 (not evaluated in this study) in the second exon of the *TERT* gene (in grey) are shown. They reside in one LD block and are part of breast cancer risk association signal 1. rs2736107 (SNP1) resides in an estrogen responsive element (ERE). The risk-associated alleles of SNP rs2736108 (SNP2) and rs2736109 (SNP3) were associated with open chromatin, while rs145533144 (SNP4) had no effect in any of the conducted analysis. rs3215401 (SNP5) reduced *TERT* promoter activity in breast cancer cell lines but the mechanism remains unclear. rs2853669 (SNP6) reduced *TERT* promoter activity in a similar manner. This polymorphism resides in an ETS binding site next to an E-box motif for c-MYC binding and ChIP experiments indicated an allele-specific effect of rs2853669 (SNP6) on GABPA binding, a transcription factor of the ETS family. An interaction of GABPA with c-MYC or other ETS family members at this position at the *TERT* promoter is unknown. The somatic mutations (orange stars) found in several cancers at position −124 and −149 create novel ETS binding sites and have been found to increase *TERT* transcription by GABPA binding in other cell lines, which activates a switch for chromatin folding.

This study supports rs3215401 and rs2853669 as having a role in *TERT* promoter regulation. Nevertheless, it is possible that other variants, which are outside the *TERT* promoter region evaluated in this study, play a role in *TERT* transcription. We also cannot discount the possibility that any variants may act in a context-dependent manner by responding to differences in growth stimuli or treatment of the cell lines. Our results do not necessarily imply that the effects of *TERT* expression on breast cancer risk are mediated through telomere length, as opposed to non-canonical functions of TERT. A large prospective study of telomere length and cancer risk, found no association with breast cancer risk [61], nor is there any support for the hypothesis that short telomeres are associated with breast cancer risk from Mendelian randomization studies [62]. Figure 4 depicts possible mechanisms of TERT transcriptional regulation and chromatin configuration by its promoter SNPs.

All six candidate variants in risk association signal 1 are located within or in close proximity to transcription factor binding sites. The strongest candidate causal variant under our test conditions, rs2853669, is surrounded by three major transcription factor binding sites, i.e. E2F, ETS and E-box motif. ChIP data indicated an allele-specific effect of rs2853669 for MYC and the ETS family member, GABPA, but not for E2F1 and the ETS-family members, ETS2 and ELF1. MYC is a strong activator of *TERT* expression in various tissues [24, 27, 28, 63]. It has been proposed that ETS2, a member of the ETS transcription factor family which is highly abundant in mammary tissues [64], cooperates with MYC in the regulation of *TERT* promoter activity in breast cancer cells [28]. Further, ETS2 may reduce *TERT* promoter activity in lung cancer cells [48] in an allele-specific manner. The possible involvement of an ETS motif was particularly intriguing, as somatic mutations in the *TERT* promoter, which create novel ETS binding sites, have been in the focus of *TERT* activation during carcinogenesis since their discovery in 2013 [44, 45] (Figure 4). Our ChIP experiments did not indicate allele-specific binding for ETS2 although there was some evidence for increased binding of the risk-associated C-allele of rs2853669 to MYC. However, whether or not MYC and/or ETS2 are silenced, rs2853669 had an allele-specific effect on *TERT* promoter in our reporter assays. These findings might imply that MYC acts in an allele-specific manner in association with transcription factors other than ETS2, such as GABPA (Figure 4). The allele specific binding of MYC and GABPA to rs2853669 in Bre80 might regulate *TERT* transcription *in vitro*, which could be investigated by assaying allelic levels of another variant, rs2736098, in the second intron of *TERT* (r2 with rs2853669 = 0.74) [41].

ETS2 knock-down did not reduce *TERT* promoter activity in any of the three mammary epithelial cell lines. This does not rule out the possibility that ETS2 can induce *TERT* expression in other cell lines or under conditions that were not tested in our study [65]. Future ChIP experiments will be required using additional cell lines and control regions in order to clarify the mechanism by which these variants act. Notably, the MDA-MB-231 cell line harbors the somatic mutation at −124 in the *TERT* promoter [66], which creates an ETS-binding site [44, 45] and might reflect synergistic or antagonistic effects of somatic mutations and polymorphisms in this cell line.

It may be possible that ETS2 binding requires additional events such as gain-of-function *TP53* mutations [67] which are not present in the three cell lines we tested. Nevertheless, the finding that rs2853669 was the most effective in down-regulating *TERT* promoter activity and was unaffected by ETS2 silencing in our analysis of these cell lines argues that it can act independently of ETS2. Besides, there are additional members of the ETS family of transcription factors which need further investigation as they might influence *TERT* expression depending on rs2853669, GABPA being the best candidate at present.

While rs2853669 had the strongest effect in our analyses, *TERT* promoter regulation appears to be very sensitive to additional sequence variation. This is indicated by our findings, that 11 additional variants masked the effect of rs2853669 in previous work [41, 42], and also by the comparable effects of rs3215401 and rs2853669 in the down-regulation of *TERT* promoter activity in luciferase assays. The variant rs3215401 does not directly reside within a known TF binding site and so its role remains elusive at this stage.

FAIRE assays implicate rs2736108 and rs2736109 in differential open chromatin. It is possible that the risk-associated alleles of rs2736108 and rs2736109 predispose to a more closed chromatin state, contributing to decreased *TERT* expression *in vivo*. Due to their effect on chromatin configuration, an effect of open chromatin may not be detectable in transient transfection-based luciferase assays and additional experiments using a native chromatin state will be needed to define the functional interaction of rs2736108 and rs2736109 with the remaining variants of the risk-associated haplotype. This may also be true for estrogen induction of the *TERT* promoter. *TERT* expression is known to be activated by estrogen stimulation [38] and rs2736107 resides in an ERE. However, we were unable to detect a significant allele- specific effect of estrogen in the induction of *TERT* promoter activity using luciferase reporter assays. Reporter assays are artificial promoter systems and may not fully recapitulate the effects of variants on native chromatin structure. Further work in model systems will be required to elucidate how additional variants in the risk-associated signals may synergize (rs3215401) or antagonize (rs2736107) in concert with rs2853669 (Figure 4).

In summary, we have identified a common polymorphism, rs2853669, as a likely promoter variant in mediating the association of a *TERT* promoter haplotype with breast cancer risk. The biological mechanism appeared to be independent of ETS2 but may involve other proteins of the ETS family, specifically GABPA. The transcriptional regulation of *TERT* expression is a limiting factor of telomerase activity, a hallmark of most cancers. Gaining insight into the effectiveness of genetic variants in the promoter of *TERT* can aid the identification of further biomarkers and novel drug targets in the prevention of breast cancer [47, 49, 68, 69].

## MATERIALS AND METHODS

### Cell lines

The normal mammary epithelial cell line Bre80 (kindly provided by Dr Lily Huschtscha; CMRI, [70]), was grown in DMEM supplemented with MEGM SingleQuot (Lonza, CC-4136) with the exception of GA-1000 (Gentamicin, Amphotericin B) and BPE (bovine pituitary extract). It was further supplemented with 5% horse serum (Thermo Scientific, 16050130) and 100 ng/ml cholera toxin (Sigma, C8052), both of which promote epithelial cell growth. The ER-positive MCF-7 (ATCC HTB22) breast cancer cell line was cultured in DMEM (Gibco 41965039) with 10% fetal bovine serum (FBS, Biochrom S0115), 110 mg/ml sodium pyruvate (Gibco 11360070) and 10 ug/ml insulin (Sigma I3536, and Actrapid, NovoNordisk, Penfill, 3 ml (100 U/ml or 3.5 mg/ml)). The ER-negative MDA-MB-231 (ATCC HTB26) breast cancer cell line was cultured in RPMI 1640 medium (Gibco, 11875093) with 10% FBS and 110 mg/ml sodium pyruvate. All cells were grown at 37°C and in the presence of 5% CO_2_. All cells were confirmed mycoplasma negative using PCR based testing (Mycoalert kit, Lonza). Cell lines were authenticated using Geneprint 10 (Promega) conforming to ATCC standard ASN-0002-2011. STR DNA profiling was a provided service from the QIMR Berghofer core facility.

For estrogen-induction assays, cells were treated with 10 nM fulvestrant (ICI 182780; Sigma, I4409) in phenol-red free DMEM (Gibco, 31053028), supplemented with 580 mg/L L-glutamine (Gibco, 25030081) 48 hours prior to treatment with 20 nM estrogen and transfection.

### Chromosome conformation capture (3C) assay

Chromosome conformation capture (3C) analysis was conducted as previously described [71] in the Bre80 and MCF-7 cell lines. Cells were cross-linked with 1% formaldehyde in order to capture DNA interactions in the physical state. Cell lysis and isolation of nuclei was performed on ice. The fixation was stopped by two washing steps with 0.125 M Glycine-PBS. Another wash with PBS was followed by cell lysis and an isolation of nuclei. Cells were incubated with fresh lysis buffer (10 mM Tris pH 7.5, 10 mM NaCl, 0.2% IGEPAL, 1 x protease inhibitors ‘Complete’, Roche 11697498001) for 30 min and cell lysis was completed with 10 strokes of a manual glass dounce homogenizer. Nuclei were isolated by centrifugation for 6 min at 800g at 4°C. Restriction enzyme cleavage was performed using 1000 U *Nco*I (New England Biolabs) in 1.2x restriction enzyme buffer (NEB buffer 2, water, 0.3% SDS, 2% Triton). After 12-15 h the restriction enzyme was heat-inactivated (80°C for 20 min in 1.6% SDS). Free ends of cross-linked DNA were re-ligated under high-dilution conditions (8 ml in total with 1% Triton, 8.7% 10x Ligase buffer (500 mM Tris pH 7.5, 100 mM DTT, 100 mM MgCl2), 0.1 mg/ml BSA (NEB B9000S), 1 mM ATP (Sigma A7699) and 40000 U T4 ligase (HC) (NEB M0202T)) and the cross-linking was reversed by proteinase K (300 ug, Astral scientific AM0706) incubation. Ligated DNA products were isolated by phenol-chloroform extraction and precipitated (0.04% Glycoblue, Ambion/Life Technologies AM9516, 120 mM sodium acetate, 50% absolute ethanol). Further, the fragments were purified using the Amicon ultra- 0.5 30K filter unit (UFC503008) according to the manufacturer's instructions. The genomic control DNA was extracted separately by the salting-out method.

Initially, we used the fragment covering the *TERT* promoter (hg19: 1286368-1297580, except for rs2736107) as the bait and then repeated the analysis with another bait in the *CLPTM1L* promoter (hg19: 1342655-1358754). Interactions between the bait promoter fragment and all other fragments along the *TERT-CLPTM1L* locus (hg19 chr5:1,249,069-1,358,885) were analyzed by qRT-PCR (Qiagen, Rotor-Gene Q) with following settings: initial denaturation at 95°C for 5 min, 55 cycles of 95°C for 45 s, 60°C for 45 s and 72°C for 60 s, final extension at 72°C for 5 min. The MyTaq HS DNA polymerase (Bioline BIO-21113) was used together with Syto9 (Life Technologies S34854) for qRT-PCR set-up. All qRT-PCR products were gel-separated in order to verify single amplicons.

Primers were designed using Primer Express (Applied Biosystems) with the following criteria: primer starts within 80-100 bp from restriction site, melting temperature between 68-70°C, 3’ GC clamp and 40-60% GC content. Primer sequences and genomic regions analyzed are shown in Supplementary Table 1 and Supplementary Figure 1.

Allele-specificity was investigated by sequencing the qRT-PCR products. Deviations of less than one-third from the heterozygous condition were considered non-significant.

### Luciferase reporter assay

Wildtype and variant haplotype *TERT* promoter sequences (3915bp) harboring all major or all risk-associated alleles of rs2736107, rs2736108, rs145544133, rs2736109, rs3215401 and rs2853669 were synthesized by GenScript and were cloned into a pGL3 reporter vector (Promega E1751). Combinations and single variants were subsequently introduced by cloning fragments containing the variant(s) of interest into the wildtype construct. The constructs were sequence verified after they were obtained from GenScript and following each completed cloning step.

Cells were harvested and reverse transfected with equimolar amounts of 300 ng *TERT* promoter luciferase reporter constructs and 50 ng of pRLTK (Promega E2241) using Lipofectamine 2000 (Life Technologies 11668027), according to the manufacturer's instructions. After 24 h the cells were lysed using the Dual Glo Luciferase Assay System (Promega E2920), according to the manufacturer's instructions. Luminescence activity was measured with Glomax (Promega) or Synergy H4 hybrid reader (BioTek). All statistical tests were performed on the log-scale, however for ease of interpretation values were back transformed for plots presented. The log-scale was used as the underlying relationship is multiplicative. All further calculations were performed on the log scale using only addition and subtraction. This includes background correction (Supplementary Figure 2) as well as statistical analysis. Data from three independent experiments were combined using a two-way ANOVA with experiment as a blocking factor. Post-hoc comparison to the wildtype construct was adjusted for multiple comparisons using Dunnett correction.

### SiRNA mediated knock-down of ETS2 and MYC

SMART pool siRNAs (negative control D-001810-10, ETS2 L-003888-00 and MYC L-003282-02) were obtained from GE Dharmacon and resuspended in 50 ul DNase- and RNase-free water for a 100 uM stock solution. Cells were harvested and reverse transfected with 20 nM siRNA using Lipofectamine 3000 (Life technologies L3000008), according to the manufacturer's instructions. After 24 h cells were harvested and reverse transfected with equimolar amounts of luciferase reporter constructs and 50 ng of pRLTK using Lipofectamine 3000. The next day, cells were lysed using the Dual Glo Luciferase Assay System, according to the manufacturer's instructions. Luminescence activity was measured with Glomax. All statistical tests were performed on the log-scale, however for ease of interpretation values were back transformed for the plots presented. Data from three or more independent experiments were analyzed using the three-way ANOVA with experiment as a blocking factor and an interaction term for silencing cell line. Multiple comparisons between groups of interest were adjusted using Bonferroni's multiple hypotheses testing correction.

### Immunoblotting

For cell lysis a cell extraction buffer was used with following ingredients: 50 mM Tris pH 7.4 (Merck), 150 mM NaCl (Merck), 2 mM ethylene glycol tetraacetic acid (EGTA; Sigma), 2 mM ethylene diamine tetraacetic acid (EDTA; Sigma), 25 mM NaF (Sigma-Aldrich), 0.1 mM Na_3_VO_4_ (Sigma), 0.1 mM phenylmethanesulfonylfluoride (PMSF; Sigma), 2 mg/ml leupeptin (Serva Feinbiochemika), 2 mg/ml aprotinin (Serva Feinbiochemika), 0.2% Triton X-100, and 0.3% Nonidet P-40 (Sigma). Cells were incubated for 30 min on ice. Separation of protein extracts was achived by SDS-PAGE and proteins were transferred via immunoblotting. Primary antibodies were rabbit anti-ETS2 (1:1000, GTX104527, GeneTex), rabbit anti-MYC (1:1000, sc-40; Santa Cruz) and mouse anti-β-actin (1:3000, A5541; Sigma-Aldrich). For detection anti-mouse IgG horseradish peroxidase-labeled secondary antibody (1:10000, NA9310; GE Healthcare) and ECL (Thermo Scientific/Pierce 32106) were used.

### Chromatin immunoprecipitation (ChIP)

ChIP experiments were conducted using the MAGnify™ Chromatin Immunoprecipitation System (Thermo Scientific 492024) following the instructions of the manufacturer. Exclusively, the normal mammary cell line Bre80 was used for ChIP as these cells represent the normal state in beast tissue. Cells were cross-linked with 1% formaldehyde followed by cell lysis. Approximately 250 000 cells were required per sample. The sonicator Bandelin Sonoplus HD 2070 generated chromatin fragments with a final size of 200-400 bp. For the immunoprecipitation with Protein A/G magnetic beads the following antibodies were used: 4 ul MYC (Cell Signaling #9402), 2 ul E2F1 (Cell Signaling #3742), 5 ul ETS2 (GeneTex GTX104527), 3 ul GABPA (Santa Cruz sc-22810-X) and 3 ul ELF1 (Santa Cruz sc-631-X). DNA was isolated, PCR-amplified and sequenced via Sanger sequencing with following primer pair: 5’-GGAGGCGGAGCTGGAAGGTGAAGG-3’ and 3’-CCAGTGGATTCGCGGGCACAGAC-5’.

The enrichment was calculated as follows: The input fraction was 10% of the chromatin used for ChIP. Consequently, to adjust the input the log2 of 10 (=3.32 cycles) was subtracted from the input Ct value and the percent of input were calculated using the formula 100*2^(Adjusted input Ct - Ct _ChIP_). Data were normalized to the negative rabbit IgG antibody (Figure 3A).

### Formaldehyde-assisted isolation of regulatory elements (FAIRE)

FAIRE was conducted as previously described [72]. In brief, breast tissue from 22 unrelated healthy female donors undergoing mammoplasty at Hannover Medical School was pulverized in liquid nitrogen and cross-linked with 1% formaldehyde followed by cell lysis. Chromatin was fragmented using the sonicator Bandelin Sonoplus HD 2070 to a final size of 200-400 bp. Input DNA was de-crosslinked by proteinase K incubation prior to phenol-chloroform extraction to isolate genomic DNA. FAIRE-enriched DNA was isolated first by phenol-chloroform extraction for the enrichment of open chromatin and incubated with proteinase K afterwards. DNA was precipitated by ethanol and prepared for genotyping via SNP-type assays (Fluidigm Inc.) using 48.48 Dynamic Genotypic IFC Arrays on the BioMark HD platform (Fluidigm Inc.) following the instructions of the manufacturer. SNP-type assays were custom designed by Fluidigm for SNPs 2, 3, 5 and 6. Cluster plots were evaluated for evidence of allelic imbalances in the FAIRE-enriched samples from individuals heterozygous for the variants of interest.

### Statistical analysis

All statistical analysis were performed using GraphPad Prism 6 apart from three-way ANOVA, which was conducted via STATA. Log-transformations were done in Microsoft Excel.

## SUPPLEMENTARY MATERIALS FIGURES AND TABLES





## Abbreviations

3C: chromosome conformation capture analysis
ChIP: chromatin immunoprecipitation
ER: estrogen receptor
ERE: estrogen-responsive element
FAIRE: formaldehyde-assisted isolation of reg-ulatory elements
GWAS: genome-wide association studies
Indel: insertion/deletion polymorphism
SNP: single nucleotide polymorphism (including germline indels)
TF: transcription factor.
