# Supplementary material for: Functional dissection of breast cancer risk-associated TERT promoter variants
Source: Oncotarget. 2017 May 26;8(40):67203–17. doi: 10.18632/oncotarget.18226 (PMC5620167; doi:10.18632/oncotarget.18226)
Supplement: Supplementary file 1 [file oncotarget-08-67203-s001.pdf]

Functional dissection of breast cancer risk-associated *TERT* promoter variants

SUPPLEMENTARY MATERIALS

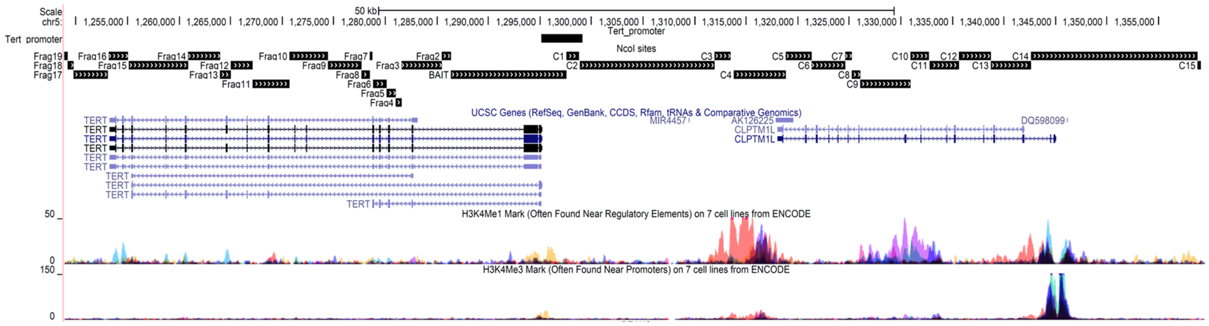

Supplementary Figure 1: UCSC genome browser session showing all fragments investigated by chromosome conformation capture analysis (3C) in the *TERT-CLPTM1L* breast cancer susceptibility locus. Fragments 2-19 cover the *TERT* gene, the BAIT fragment covers the *TERT* promoter and the fragments C1-C15 cover the *CLPTM1L* gene and the region between the two genes. For every fragment a primer was designed and named accordingly either TERT NcoI frag 2-19, *TERT* promoter bait or CLPTM1L NcoI frag 1-15. CLPTM1L NcoI frag 14 was used as bait in the reciprocal experiment.

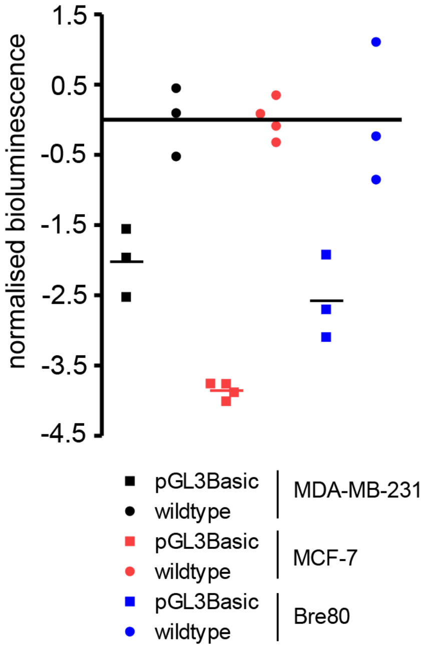

Supplementary Figure 2: Comparison of the *TERT* promoter wildtype construct versus the empty pGL3Basic vector used in luciferase assays. Values are presented in log-scale and were normalised to the wildtype construct in MDA-MB-231, MCF-7 and Bre80.

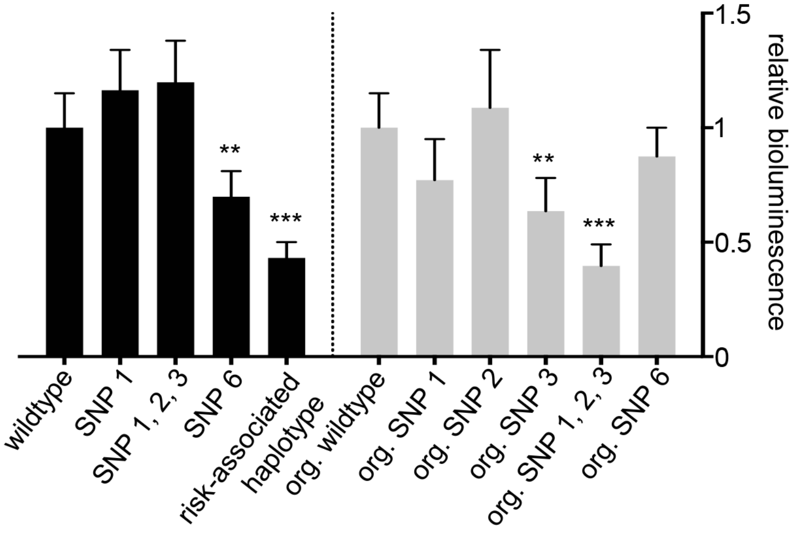

Supplementary Figure 3: Comparison of the effect of SNPs 1, 2, 3 or 6 using the *TERT* promoter construct from our study (left side) versus the original *TERT* promoter constructs previously used harboring second-site-mutations (right side). MCF-7 cells were reverse transfected with *TERT* promoter constructs as indicated in the graph. Data are shown for three experiments. Values were normalised to wildtype. Error bars represent 95% confidence interval from at least 3 experiments. Two-way ANOVA multiple comparison tests were performed, using Dunnett correction (* P<0.05, ** P<0.01, ***P<0.001). All statistical analysis were performed in log-scale, values were back-transformed for plots presented.

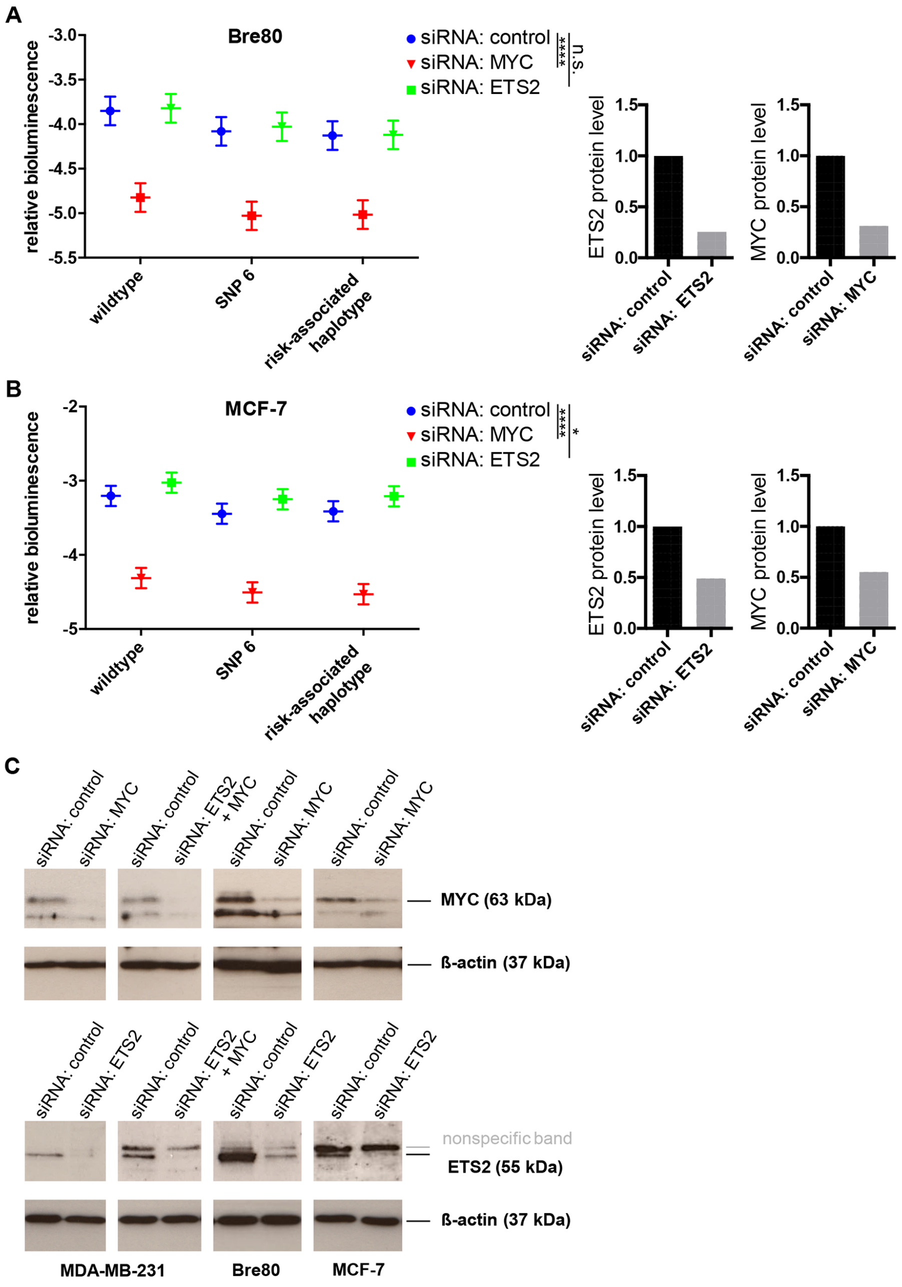

Supplementary Figure 4: *TERT* promoter activity upon MYC or ETS2 silencing and knock down efficiency. (A, B) Cells were reverse transfected with siRNA: control or ETS2 or MYC or ETS2 and MYC and 24h later reverse transfected with *TERT* promoter constructs. The wildtype construct was compared to SNP6 and the risk-associated haplotype in the reference cell line Bre80 **(A)** and the ER+ cell line MCF-7 **(B)**. Knock-down efficiency is indicated next to graphs. Data are shown for two experiments. ANOVA was used to assess the effect of group, siRNA and the interaction of group and siRNA. Experiments were performed on separate days and results were combined by including a blocking factor into the ANOVA. The figures represent the estimated marginal effect (with 95% confidence interval) of each treatment combination after accounting for the average difference between separate days. Multiple comparisons between groups of interest were defined via contrasts. P-values were adjusted using Bonferroni's multiple hypothesis testing adjustment. All statistical analysis were performed in log-scale. **(C)** Knock-down efficiency was determined by western blot analysis for MDA-MB-231, Bre80 and MCF-7. ß-actin served as loading control. ETS2 antibody detected an unspecific band above ETS2. Membranes were cut horizontally between 50kDa and 37kDa marker bands to detect MYC/ ETS2 and ß-actin simultaneously.

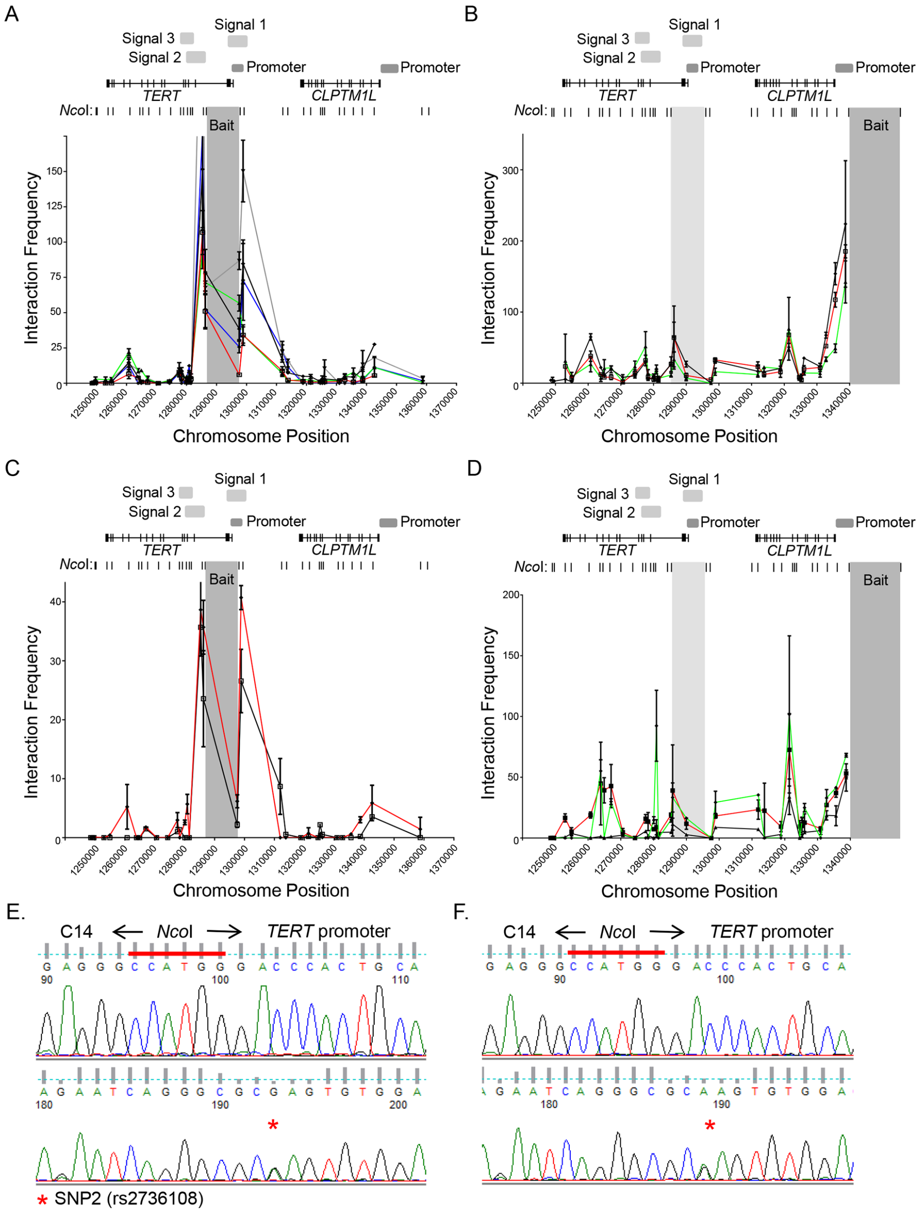

Supplementary Figure 5: Chromosome conformation capture (3C) analysis demonstrated no significant interaction between the *TERT* or *CLPTM1L* promoter with proximal regions at the *TERT-CLPTM1L* locus. For 3C analysis libraries were generated using *Nco*I. Genomic regions covered are indicated. The bait fragment (dark grey) covered the *TERT* promoter **(A, C)** or the *CLPTM1L* promoter **(B, D)**, respectively. The light grey area **(B, D)** indicates the position of the previous bait fragment. Interactions were analyzed in MCF-7 (A with n=5, B with n=3,) and in the reference line Bre80 (C with n=2, D with n=3). Colored lines in the graph indicate individual experiments. Interaction frequencies were calculated as $(PE_{target})^{CT\ target}$. Values were normalized to GAPDH. The interaction between the two fragments covering the TERT promoter and the CLPTM1L promoter (C14) was verified by Sanger Sequencing in MCF-7 **(E)** and Bre80 **(F)**. Asterisks mark position of SNP2 (rs2736108).

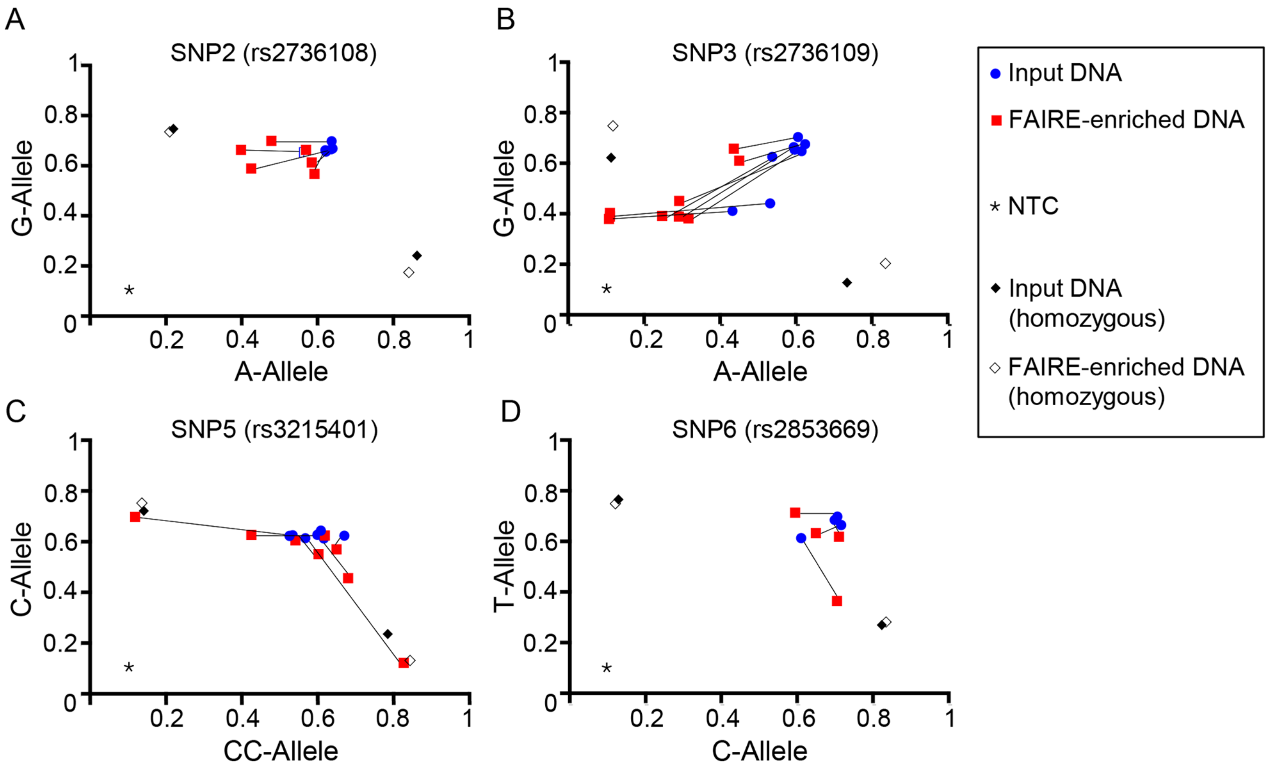

Supplementary Figure 6: FAIRE-analysis at the *TERT* promoter SNPs 2, 3, 5 and 6. Breast tissue was pulverized and cross-linked followed by cell lysis and DNA isolation. SNP-type genotyping was performed, and eight patients heterozygous for the *TERT* promoter SNP haplotypes were investigated by comparison of input and FAIRE-enriched samples. For SNP2 **(A)** and SNP3 **(B)** the signal of the minor G-allele decreases after FAIRE-enrichment, whereas signals for each allele remained equal in cross-linked samples for SNPs 5 **(C)** and SNP6 **(D)**. Means of technical duplicates are shown.

Supplementary Table 1: Primer sequences for qRT-PCR for chromosome conformation capture analysis (3C) with bait 1 covering the *TERT* promoter and bait 2 covering the *CLPTM1L* promoter for the reciprocal experiment.

See Supplementary File 1

Supplementary Table 2: Additional variants in the original *TERT* promoter construct used in previous studies* identified by sanger sequencing

Variant [ref/alt]	Chr. position	SNP	MAF	Predicted changes in TF motifs
A / T	1298806	-	-	-
G / A	1298782	rs2736106	0.38	20
A / G	1298485	-	-	-
A / G	1297922	rs7449190	0.14	-
G / A	1297714	-	-	-
T / C	1297459	-	-	-
A / G	1297258	rs6554754	0.10	2
A / G	1296748	-	-	-
T / C	1296486	rs2735940	0.47	3
T / C	1296072	rs7712562	0.12	4
A / G	1295893	-	-	-

Ref.: reference allele; alt.: alternative allele; chr.: chromosome; SNP: single nucleotide polymorphism; MAF: major allele frequency; TF: transcription factor.

* Previous studies: Beesley et al., 2011 and Bojesen et al., 2013.
