# Supplementary material for: Functional dissection of breast cancer risk-associated TERT promoter variants
Source: Oncotarget. 2017 May 26;8(40):67203–17. doi: 10.18632/oncotarget.18226 (PMC5620167; doi:10.18632/oncotarget.18226)
Supplement: Supplementary file 2 [file oncotarget-08-67203-s002.doc]

**Supplementary Table 1: Primer sequences for qRT-PCR for chromosome conformation capture analysis (3C) with bait 1 covering the *TERT* promoter and bait 2 covering the *CLPTM1L*** promoter for the reciprocal experiment

| **Primer** | | **Orientation** | | **Chromosome position** | | **Sequence** | **Bait** | |  |
| --- | --- | --- | --- | --- | --- | --- | --- | --- | --- |
| 2-Tert Frag 19 | Forward | | 1248711 | | 1249194 | | CCCTGAAGCTTCAGAGTCCTTGTCCAGC |  | |
| 2-Tert Frag 18 | Reverse | | 1249200 | | 1249752 | | GCAATGTGATGGGACAGACCACCTCTGC |  | |
| 2-Tert Frag 17 | Reverse | | 1249758 | | 1253121 | | CCCTGAACCTCGTTTTCCCATCAAGTGC |  | |
| 2-Tert Frag 16 | Forward | | 1253189 | | 1255057 | | CCTTCGGGCCTGGACTTACTGTTATGTCTTC |  | |
| 2-Tert Frag 15 | Reverse | | 1255130 | | 1260866 | | CCTAATGTGCACTGCATAGACACCACTGTATGC |  | |
| 2-Tert Frag 14 | Forward | | 1260872 | | 1263976 | | GGAATGTCAGACACAGGTGCCTGCC |  | |
| 2-Tert Frag 13 | Reverse | | 1263982 | | 1265010 | | GGGCAGATGATGAGTGCACAAACACG |  | |
| 2-Tert Frag 12 | Forward | | 1265016 | | 1267128 | | CAGGACAGGTAGGTGAGCATGCAAGAACC |  | |
| Tert Frag 11 | Reverse | | 1267134 | | 1270687 | | GGGCATCTAGGAGAAAACAGGCAAAGTCG |  | |
| Tert Frag 10 | Reverse | | 1270693 | | 1274425 | | GCATGTACAGTGGATCAAGGTTCTTCTTCATTAAGG |  | |
| Tert Frag 9 | Reverse | | 1274431 | | 1277654 | | CTCATTCCTGTCCCTGTCGTGTGACCC |  | |
| 2-Tert Frag 8 | Forward | | 1277660 | | 1278538 | | GCCATTCATGGACAGAACGCACAGC |  | |
| Tert Frag 7 | Reverse | | 1278544 | | 1278783 | | ACCTGTGCCCGGGTGTAGAAAGTCACC |  | |
| Tert Frag 6 | Reverse | | 1278789 | | 1280154 | | CCTCCACTCACACAGGTGGATGTGACG |  | |
| 2 Tert Frag5 | Reverse | | 1280160 | | 1280993 | | GCCAGAACGTTCCGCAGAGAAAAGAGG |  | |
| 2 Tert Frag4 | Reverse | | 1280999 | | 1281611 | | CCTTTTCTACTCTGCTGGGCCTGCG |  | |
| Tert Frag 3 | Reverse | | 1281617 | | 1285454 | | GTTCCACCATGAGCTAACTTCTAGGTGGCTGC |  | |
| 2 Tert Frag2 | Forward | | 1285466 | | 1286362 | | CCACGTGGTGCTCCAGACACTCACG |  | |
| Tertp Frag 1 | Forward | | 1286368 | | 1297580 | | GGCTCAATCCCAGTAGAGTAGGAGGGATGG | Bait 1 (*TERT* promoter) | |
| CLPTM1L Frag1 | Forward | | 1297586 | | 1298819 | | CCATAACTGAGCCAATACCATCAGAACCAGG |  | |
| CLPTM1L Frag2 | Forward | | 1298870 | | 1311929 | | GGGCAGGTCTGCAGTCAGGTTTGTACC |  | |
| CLPTM1L Frag3 | Forward | | 1311935 | | 1313487 | | GGTTTTCAACGCCGCTAGTCAGCAGG |  | |
| CLPTM1L Frag4 | Reverse | | 1313812 | | 1318832 | | GCTCAACAAGCCCTGCATGCTTCC |  | |
| CLPTM1L Frag5 | Reverse | | 1318838 | | 1321388 | | CTGATGAGACGTGGTCCCTGAACACAGC |  | |
| CLPTM1L Frag6 | Forward | | 1321394 | | 1324581 | | CCAACAGCAATGGGGAAAGGTGACG |  | |
| CLPTM1L Frag7 | Reverse | | 1324587 | | 1325271 | | GGCTCGCAGAGACTCATTTCCCAGTCC |  | |
| CLPTM1L Frag8 | Reverse | | 1325277 | | 1326024 | | GCCCACGGATGGACCTTGATTGAGG |  | |
| CLPTM1L Frag9 | Forward | | 1326030 | | 1330958 | | CTGGATTTGTTTCCTCCCCAGCTGTACC |  | |
| CLPTM1L Frag10 | Reverse | | 1330964 | | 1332747 | | CCGTGTTCTGCGTTGTTGTCTCCTAACC |  | |
| CLPTM1L Frag11 | Forward | | 1332767 | | 1335632 | | CGCTAGCAGTGCCTGGTCTGTTTTGC |  | |
| CLPTM1L Frag12 | Reverse | | 1335638 | | 1338725 | | GAGGCACTTTGCCCATGTGCTGC |  | |
| CLPTM1L Frag13 | Reverse | | 1338731 | | 1342649 | | CCAGCTCTTTCCCACTTCCTCATTGAGG |  | |
| CLPTM1L Frag14 | Forward | | 1342655 | | 1358754 | | GGAAGGGTTTTGCCAGATGCCTGG | Bait 2 (*CLPTM1L* promoter) | |
| CLPTM1L Frag15 | Reverse | | 1358760 | | 1359074 | | GTGGCCTGTGTCCTCCAATGTCAGG |  | |

Frag: fragment.
